# Cell Atlas technologies and insights into tissue architecture

**DOI:** 10.1042/BCJ20190341

**Published:** 2020-04-27

**Authors:** Anna Wilbrey-Clark, Kenny Roberts, Sarah A. Teichmann

**Affiliations:** Wellcome Sanger Institute, Cambridge, U.K.

**Keywords:** cell atlasing, single-cell RNA sequencing, smFISH, spatial

## Abstract

Since Robert Hooke first described the existence of ‘cells’ in 1665, scientists have sought to identify and further characterise these fundamental units of life. While our understanding of cell location, morphology and function has expanded greatly; our understanding of cell types and states at the molecular level, and how these function within tissue architecture, is still limited. A greater understanding of our cells could revolutionise basic biology and medicine. Atlasing initiatives like the Human Cell Atlas aim to identify all cell types at the molecular level, including their physical locations, and to make this reference data openly available to the scientific community. This is made possible by a recent technology revolution: both in single-cell molecular profiling, particularly single-cell RNA sequencing, and in spatially resolved methods for assessing gene and protein expression. Here, we review available and upcoming atlasing technologies, the biological insights gained to date and the promise of this field for the future.

## Introduction

Cell atlasing initiatives have expanded rapidly in recent years; with the goal of identifying at the molecular level, and therefore aiming to better understand, cell types in different organisms and model systems. These initiatives include the Human Cell Atlas and allied projects such as the Mouse Cell Atlas/Tabula Muris and the Malaria Cell Atlas among others [1–3]. The number of publications aiming to comprehensively identify new cell types and the resulting volume of raw data made publicly available has increased substantially in the last decade. Therein lies the challenge and benefit of such initiatives — the datasets generated are generally very large resources that will require the input of many specialists across the scientific community to analyse and validate; therefore, we are unlikely to understand the full benefits of these studies for several years. However, even in the first stages of analysis, these projects have given significant new biological insights, outlined at the end of this review. These data also offer huge potential for medicine, drug discovery and diagnostics through a more detailed understanding of cell types, basic biological processes and disease states.

A major driver behind the expansion of atlasing initiatives in recent years is the advent of single-cell RNA sequencing technology, particularly massively parallel sequencing, which allows the generation of whole-transcript (mRNA) data from thousands of cells quickly and easily. However, these technologies require tissues to be dissociated to single cells, a process that is usually biased and loses the cells’ physical context. Given that each cell's position within a tissue is often critical for its function, technologies are emerging to understand the spatial location of cells within tissue architecture. This review will focus upon these two classes of atlasing technology: large-scale single-cell sequencing advances and spatially resolved methods.

## The single-cell RNA sequencing revolution

Assessment of gene expression in tissues and model systems is a valuable way of understanding their cellular composition and function, and the changes that occur during disease or drug treatment. For many years, PCR, microarray and ‘bulk’ RNA sequencing required hundreds or thousands of cells to be pooled, giving a population-level view that could not distinguish rare cell populations or whether gene expression changes were due to a strong response in a few cells or weaker response across all cells. Single-cell qPCR first allowed these assessments in individual cells, but was limited in the number of genes detectable [4–6]. In 2009, Tang et al. [7] published the first whole-transcriptome single-cell mRNA sequencing on mouse blastomeres. Over the following 5 years, many new methods were developed and improved: notably STRT-seq [8, 9], SMART-seq [10], CEL-seq [11] and SMART-seq2 [12]. These technologies sequenced mRNA 5′ ends (STRT-seq), 3′ ends (CEL-seq) or full-length mRNA (SMART-seq/SMART-seq2) and either used *in vitro* transcription (CEL-seq) or PCR-based amplification (STRT-seq/SMART-seq/SMART-seq2). *In vitro* transcription provides linear amplification but is time-consuming; PCR-based amplification is quicker but suffers from bias due to its exponential nature. These initial approaches were low-throughput and labour-intensive, run on a few dozen manually picked cells or on flow-sorted 96 well plates.

**Figure 1.**
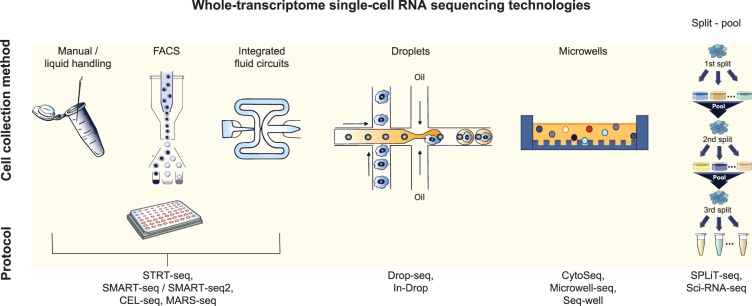
Single-cell RNA sequencing technologies. Summary of methods for compartmentalising single cells for scRNAseq (top row) and the technologies that use them (bottom row; see also Table 1). Images adapted from [1,18].

In 2014, MARS-Seq was published, which used liquid handling in 384 well plates to massively increase the number of cells that could be sequenced to over 1000 [13]. Thereafter followed nanowell, droplet and *in situ* techniques, all of which used barcoding to mark transcripts coming from the same cell, thus making it possible to sequence tens of thousands of cells in parallel [14–20]. As well as per-cell barcodes, all of the larger-scale techniques incorporate unique molecular identifiers (UMIs); random 4–8 bp sequences that label each individual mRNA molecule in that cell, allowing individual molecule counting to compensate for PCR bias. To achieve high cell yield in a cost-effective manner, these methods rely on pooling the bead-bound mRNA or first-strand products from all cells and sequencing only the 5′ or 3′ end of transcripts at low depth, therefore, losing the ability to study splice isoforms and SNPs, which is feasible with full-length data [21]. A summary of scRNAseq methods is presented in Table 1 and Figure 1.

**Table 1.** scRNAseq technologies

Technology	Ref	Cell separation method	PCR or IVT?	3′, 5′ or full-length data?	UMIs?	Maximum cell diameter (μm)	Throughput
STRT-seq	[8,9]	Manual/FACS	PCR	5′	N	Dependent on cell collection method	Low (10–100 cells)
SMART-seq/SMART-seq2	[10,12]	Manual/FACS	PCR	Full-length	N	Dependent on cell collection method	Low (10–100 cells)
CEL-seq	[11]	Manual/FACS	IVT	3′	N	Dependent on cell collection method	Low (10–100 cells)
MARS-seq	[13]	FACS	IVT	3′	Y	Dependent on cell collection method	Medium (1000 cells)
Cytoseq	[14]	Nanowell (gravity)	PCR	3′	Y	<30*	High (10 000 cells)
Seq-well/Seq-well S^3	[15,22]	Nanowell (gravity)	PCR	3′	Y	<45*	High (10 000 cells)
Microwell seq	[1]	Nanowell (gravity)	PCR	3′	Y	<30*	High (10 000 cells)
Drop-seq	[17]	Droplet	PCR	3′	Y	<125*	High (10 000 cells)
In-Drop	[16]	Droplet	IVT	3′	Y	<60*	High (10 000 cells)
SPLiT-seq	[18]	*In situ* barcoding	PCR	3′	Y	Unrestricted	High (10 000+ cells)
sci-RNA-seq	[19]	*In situ* barcoding	PCR	3′	Y	Unrestricted	High (10 000+ cells)

Summary of main published scRNAseq methods. PCR, polymerase chain reaction; IVT, *in vitro* transcription; UMIs, unique molecular identifiers.

* Well/droplet size; must accommodate cell and bead.

Nanowell methods such as Cytoseq [14], Seq-well [15], Seq-well S^3 [22] and Microwell-seq [1] rely on gravity to load cells with a Poisson distribution into picolitre-sized wells. Oligo-dT beads with UMIs, cell barcodes and a PCR handle are then loaded into all wells. As nanowells are often transparent, they allow the opportunity to observe the captured cells under the microscope, such that cell morphology, doublet rate and sometimes viability or other stainings can be assessed. It is also sometimes feasible to ‘wash-out’ chips if too many cells (and therefore doublets) are loaded. Stronger lysis buffers can be used than with droplet or plate-based technologies [15] (with some exceptions, for example, cells can be lysed in the harsh lysis buffer RLT followed by mRNA pulldown and SMART-seq2 in plates [23]). However, it is not usually possible to image all cells without fast microscope platforms adapted for the chips and currently methods that allow linkage between a cell image and its associated barcode are rare. Well sizes are typically in the order of 30–50 μm which limits the maximum cell size that can be loaded, making the majority of the gravity-fed microwell platforms unsuitable for large cells such as 100 μm cardiomyocytes or oocytes.

Droplet-based methods including Drop-seq and In-Drop [16,17,24] also rely on beads covalently linked to oligo-dT, UMIs, cell barcode and PCR handle for 3′ end sequencing. However, instead of gravity-loading into wells, cells and beads are captured with Poisson distribution into the water in oil droplets (emulsion). These serve as mini reaction vessels in which the first-strand synthesis can take place, before pooling by emulsion breakage, second-strand synthesis and amplification/library preparation. These systems do require more specialist equipment than microwell platforms and it is not usually possible to image the cells within the droplets. The droplet size also limits the maximum cell size that can be captured. However, commercialisation of droplet-based sequencing, especially launch of the 10× Genomics Chromium platform, has made it a fast, easy-to-use and popular method for sequencing thousands of single cells in parallel and advances are being made in incorporating a wider range of cell sizes.

The most recent scRNAseq techniques use *in situ* barcoding [18,19], in which cells are labelled with multiple barcodes by pooling and splitting the cells at each stage of RT/ligation/PCR, resulting in up to a million potential barcode combinations and therefore the possibility of labelling tens to hundreds of thousands of cells per sample. This method has the advantage of not requiring specialist commercial equipment, multiplexing many samples at a time and is compatible with fixed cells or nuclei, whereas other methods typically use fresh cell suspensions.

As published scRNAseq methods advance, these are being rapidly commercialised (Table 2). One of the first marketed systems was the Fluidigm C1, which used integrated microfluidic circuits to capture, lyse and reverse transcribe up to 96 single cells using a full-length mRNA Smart-Seq2-based protocol. Different cell sizes were captured on different chips and with suitable equipment, each cell could be imaged and this information linked to its transcriptomic data. As single-cell sequencing expanded in scale, this system was adapted to capture up to 800 cells using 3′ end sequencing.

**Table 2.** Commercial scRNAseq platforms

Platform	Supplier	Cell separation method	PCR or IVT?	Library prep method	Uses UMIs?	3′, 5′ or full-length data?	Maximum cell diameter (µM)	Throughput
C1	Fluidigm	IFCs	PCR	Template switching	Protocol dependent	Full length, 3′, 5′	25	Low–medium (10–800 cells)
Chromium	10× Genomics	Droplet	PCR	Template switching	Y	3′ or 5′	30–60*	High (10 000)
Nadia/Innovate	Dolomite Bio	Droplet	PCR	Template switching	Y	3′	40/100*	High (10 000)
C-prep Genesis	CelSee	Nanowells = gravity or forced	PCR	Template switching	Y	3′	30*	High (10 000+)
iCell8	Takara	Nanowells - nanodispensing	PCR	Template switching	Y	Full-length	Unrestricted	Medium (1000)

Summary of major commercially-available scRNAseq platforms. PCR, polymerase chain reaction; IVT, *in vitro* transcription; UMIs, unique molecular identifiers.

* Well/droplet size; must accommodate cell and bead.

With the advent of droplet sequencing emerged the 10× Genomics Chromium [25] platform and Dolomite Bio's Drop-seq-based Nadia platform. These systems typically capture 5000–10 000 cells per channel depending on cell concentration loaded (eight channels can be run in parallel on both) in droplets of 50–60 µm on the 10× Chromium and up to 100 µm on the Nadia Innovate. Both platforms utilise a template switching, 3′ end-counting protocol in which individual transcripts are labelled with UMIs. In spite of high per-sample costs, the fast robust workflows and excellent cell yields have made droplet scRNAseq a popular choice in recent years.

Two commercial players in whole-transcriptome microwell-sequencing are the CelSee platform and Takara's iCell8. CelSee's Genesis system is a gravity-based technology in which cells are loaded using Poisson distribution into 30 μm wells [26]. Though still under development, this system aims to generate 3′ end data using a PCR-based template switching mechanism and to allow imaging and capture of rare cells. Takara's iCell8 [27] uses liquid handling to distribute cell suspensions with Poisson distribution across a 5071 nanowell chip. The large well size on the iCell8 allows cells such as cardiomyocytes to be captured and the system has low magnification (4×) fluorescence imaging capability, allowing assessment of doublets, live/dead staining or other fluorescent stains and linkage between this image and the Smartseq2 full-length cell transcriptome generated. However, the number of cells captured is in the range of 1000–1800, rather than the thousands reported for gravity-fed microwell and droplet technologies. Indeed, in general, there is a trade-off: technologies with lower cell capture rates (hundreds of cells) often allow full-length transcript sequencing suitable for splice isoform analysis and the possibility of linking the cell barcode and image of the cell, whereas higher-throughput technologies (thousands of cells) use end-counting methods and either have no cell imaging capability or when cells can be imaged, currently it is not possible to link an image to a cell transcriptome. Therefore, the choice of system for scRNAseq is very much dependent on the cell types used and the data the end user requires.

In addition to gene expression analysis, many of the commercial scRNAseq platforms now offer additional protocols such as chromatin accessibility (ATAC-seq), V(D)J/T-cell receptor (TCR) profiling of immune cells [V(D)J recombination occurs in developing lymphocytes, resulting in immunoglobulin and TCR diversity], the ability to incorporate antibody staining and to capture nuclei rather than cells. The advantage of single-nucleus RNA sequencing (snRNAseq) [28–34] is that it does not require enzymatic dissociation, so the cell types recovered are more representative of the original tissue and suffer less from transcriptional artefacts. Use of frozen samples as opposed to the fresh material required by scRNAseq techniques also allows access to archived material. Lack of cytoplasmic mRNAs does result in lower mRNA content in nuclei than cells and fewer detectable genes, but in spite of this it is often possible to identify the same cell types detected in scRNAseq [28,29]. Currently snRNAseq is following a similar progression to scRNAseq; moving from low-throughput formats [33,34] to massively parallel sequencing (DroNc-seq [29]). Single-nucleus sequencing has thus far been applied largely to the brain, but larger-scale studies ongoing should demonstrate whether it is effective in a wider range of tissues.

The next step in the profiling of dissociated single cells and nuclei is likely to be an increase in multi-omics technologies (reviewed in [35,36]). Already methods exist for profiling genomic DNA and mRNA (G&T-seq) or transcriptome, ATAC-seq and methylation state together in single cells [37–43]. This offers opportunities to reconstruct cell lineages by tracking DNA mutations [44] and provides insights into cell state. The use of antibodies conjugated to DNA barcodes that can be readout through sequencing is also increasing and allows profiling of hundreds of proteins across thousands of single cells or nuclei, with paired whole-transcriptome data [45,46].

Indeed, highly multiplexed proteomics is particularly challenging due to the requirement for good quality, specific antibodies. However, understanding protein expression is essential given that proteins are the major biological effector molecules in the cell. Methods such as mass cytometry by time-of-flight (CyTOF) and FACS allow profiling of dozens of proteins using antibodies labelled with heavy-metals or fluorophores [47,48], but such dissociated methods lose information on subcellular location, which can be critical for gene-product function. Protein immunostaining of tissue sections addresses this but is low-throughput. Some large-scale atlasing initiatives are tackling this challenge, such as the Human Protein Atlas [49,50].

One issue for all of the methods discussed above is that they require tissue or sample dissociation. A high-quality single-cell suspension should have good cell viability, absence of debris and an accurate representation of the cell types present in the original sample, but in reality, achieving this is often problematic (see technical challenges of atlasing reviewed in Hon et al. [51,52]). The majority of publications currently use a combination of mechanical and warm enzyme treatment over a prolonged period, which can itself induce stress-related transcriptional changes [53]. Some protocols aim to overcome this by using enzymes that work at cold temperatures [54,55]. A variety of fixatives or other preservation agents have also been trialled, although the majority of these were tested on single-cell suspensions post dissociation rather than intact tissue pieces [56–60]. Warm ischaemic time also creates transcriptional changes [51]; there is evidence that this can be abrogated by rapid cold storage in hypothermic preservation media [61], but this does not resolve the issues introduced by dissociation. Therefore, methods to preserve intact specimens that allow the generation of high-quality, transcriptionally accurate single-cell suspensions, are needed.

A solution is not to dissociate tissues at all. Indeed, understanding the tissue context of cell types is a critical component of atlasing, as the location is often related to function; tissue dissociation loses that context and can cause loss of specific cell populations. Small pools of cells from tissue sections can be profiled at whole-transcriptome level using laser capture microdissection [62]. A promising alternative is that of imaging and/or sequencing-based spatially resolved approaches. These methods have traditionally been used as validation tools to demonstrate, for example, the existence of new cell types predicted by scRNAseq. However, they are rapidly maturing and beginning to reach a scale appropriate for hypothesis-generating cell atlasing themselves.

## Spatial technologies: putting single cells in tissue context

**Figure 2.**
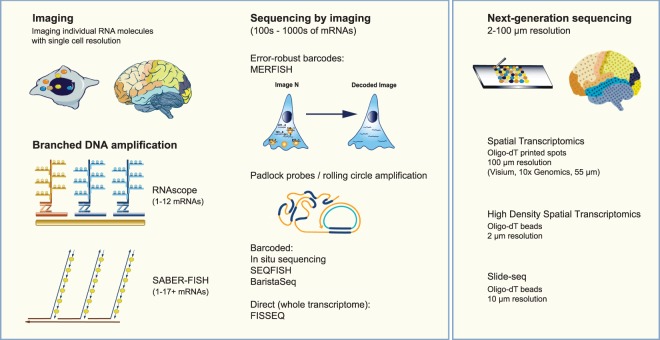
Spatially resolved methods for RNA analysis. RNA molecules may be imaged at single-cell or sub-cellular resolution using low-plex branched DNA methods (RNAScope, SABER-FISH), or higher-plex sequencing by imaging methods. Among the latter, only FISSEQ allows direct sequencing of mRNA targets without requiring prior knowledge of the RNA sequence. Other methods (MERFISH, *in situ* sequencing, SEQFISH, Barista-seq) require the use of transcript-specific probes. NGS-based methods, which use oligo-dT covalently linked to glass slides (Spatial Transcriptomics, Visium) or beads (High-Density Spatial Transcriptomics, Slide-seq) give a lower resolution view of mRNA expression patterns but do not require probe design. Images adapted from [63–67].

One of the most long-standing methods for assessing RNA distribution in tissues is smFISH. DNA probes are hybridised to thin fresh frozen or fixed tissue sections, usually followed by signal amplification, and individual RNA molecules read-out by imaging (for a summary of methods see Table 3 and Figure 2). These methods have the advantage of high sensitivity compared with scRNAseq [78] and excellent spatial resolution, allowing visualisation of individual cells and even sub-cellular structure, while retaining information on each cell's position within the tissue. SmFISH methods vary in scale considerably, from single-plex or lowly multiplexed (RNAscope, SABER-FISH, osmFISH, PLISH [63,64,68,69,73,79]) to those measuring hundreds or thousands of mRNAs through the use of imageable barcodes (MERFISH; *in situ* sequencing; seqFISH; BaristaSeq [64–66,70–72,80–82]). Methods are now starting to be developed that aim to deliver whole-transcriptome, spatially resolved measurement of mRNAs in tissue sections such as RNA SPOTs (a modification of SeqFISH; [72]) and FISSEQ, which uses *in situ* imaging-based sequencing of RNA molecules directly [74].

**Table 3.** Spatially resolved methods

Protocol	Ref	Plex	Underlying technology	Spatial resolution	Readout
RNAscope	[63]	12	Branched DNA/probes. Non-barcoded	Single cell	Imaging
SABER-FISH	[64]	1–17+	Primer exchange reaction	Single cell	Imaging
osmFISH	[68]	1–33+	Probes. Non-barcoded, unamplified, cyclic	Single cell	Imaging
PLISH	[69]	1–8+	Proximity ligation/RCA	Single cell	Imaging
MERFISH	[66,70]	10 000	Barcoding and sequential imaging	Single cell	Imaging (barcodes)
*In situ* sequencing	[65]	256+	Padlock probes, RCA	Single cell	Imaging (barcodes)
seqFISH+, RNA SPOTs	[71,72]	10 000	Barcoding and sequential imaging	Single cell	Imaging (barcodes)
BaristaSeq	[73]	10 000+	Padlock probes, RCA	Single cell	Imaging (barcodes)
FISSEQ	[74]	Whole transcriptome	RCA; SOLiD sequencing	Single cell	Sequencing by imaging
Spatial Transcriptomics/10× Genomics Visium	[75,76]	Whole transcriptome	Printed oligo-dT spots	100 µM/55 µM	NGS
High-Density Spatial Transcriptomics	[77]	Whole transcriptome	Oligo-dT beads on slide	2 µM	NGS
Slide-seq	[67]	Whole transcriptome	Oligo-dT beads on slide	10 µM	NGS

Summary of published methods for assessing transcript localisation in tissue sections. RCA, rolling circle amplification.

All of these methods require expensive, specialist imaging equipment and can be challenging to implement due to spectral overlap between fluorophores, the optical diffraction limit of microscopy, and the challenges of tissue autofluorescence. Methods offering high multiplexing use multiple rounds of staining and imaging: these tend to be time- and labour-intensive, and require the non-trivial ability to wrangle large image datasets as well as accurately register both fiducial features such as nuclei and single-molecule spots. Tissues with cells that are densely packed, such as glandular epithelia of the endometrium or granule layers of the cerebellum, or that exhibit complex morphologies, such as neurons, compound these challenges. SpaceTx/STARFISH has been set up to address exactly these issues. Until high-plex methods become more accessible both experimentally and computationally, lower-plex protocols will remain the most prevalent, and are currently used extensively for validation of scRNAseq data sets. This is aided by automation of some low-plex protocols such as RNAscope on slide-staining instruments [83,84], but this requires many tissue sections and is expensive. Although a trade-off between throughput and sensitivity currently limits the scope, the ability to localise and quantify hundreds or thousands of mRNA molecules within tissue sections allows precise validation of many cell types and their locations in parallel; a scale in line with the volume of scRNAseq data generated by atlasing projects. Commercial systems that deliver this are needed and will have huge potential if the aforementioned challenges can be overcome.

While use of smFISH for validation of scRNAseq is valuable, most spatial technologies lag behind the whole-transcriptome interrogation of dissociative methods, a key element behind the power of atlasing in generating data in a hypothesis-free manner without prior knowledge of cell type markers. Aside from FISSEQ, spatial imaging methods to date cannot deliver hypothesis-free whole-transcriptome scale data combined with single-cell resolution. However, whole-transcriptome sequencing-based spatial technologies that do not require such complicated imaging equipment as smFISH-based approaches are now beginning to emerge.

In 2016, Ståhl et al. [75] published ‘spatial transcriptomics’, a method in which thin fresh-frozen tissue sections are placed over a grid of 100 μm oligo-dT spots, each spot having a unique barcode. The tissue is stained with haematoxylin and eosin to assess histology and localise the oligo spots, before being permeabilised and used to prepare sequencing libraries. The oligo-dT spot barcodes are used to assign each read to its location in the tissue, producing whole-transcriptome data with spatial resolution. Hundred micrometer resolution is useful for identifying gene expression differences between gross anatomical structures in tissues. However, for cell type profiling of complex tissues in which a 100 μm area can cover diverse cell types, it is not sufficient. Recent commercial systems and academic publications have increased this resolution by reducing feature sizes to 55 μm (10× Genomics Visium, commercial, [85]) or 2–10 μm (academic, [67,75,77]). RNA diffusion from the permeabilised cells has been demonstrated to be ∼2 μm [75]. Therefore these technologies, though in their infancy, are beginning to reach single-cell scale. This opens up the prospect of generating hypothesis-free data and new cell type prediction directly *in situ*, where the location of each cell type and its neighbours are known.

Of course, as the size of features on spatial arrays is reduced, the ability to capture mRNAs and therefore sensitivity, is also reduced. However, these methods do generate data in agreement with scRNAseq. Lower-throughput smFISH-based methods are more sensitive, localising individual mRNA molecules with sub-cellular resolution at greater than 90% detection efficiency. Therefore there is clear value in all of these technologies for atlasing initiatives.

Another technology utilising a next-generation sequencing (NGS) read-out while focusing upon areas of interest identified according to spatial features is the NanoString GeoMX Digital Spatial Profiler [86]. By permitting the user to select regions of interest according to RNAscope or immunohistochemical staining, the GeoMX DSP allows interrogation of around 100 proteins or up to 1800 mRNAs within spatially resolved features and cell populations (and a whole-transcriptome assay targeting ∼18 000 genes is to be released). Tags attached to oligonucleotide probes or antibodies are cleaved by highly refined patterns of UV light, directed by a digital mirror device module with ∼1 μm^2^ resolution, and collected via a micro-capillary system; following collection these tags are quantified using the NanoString nCounter platform or NGS. While it currently lacks single-cell resolution — with a recommended capture of >10 cells per feature for protein detection and 50–200 for RNA — this technology offers great flexibility in choosing these regions, with custom geometric and segmentation algorithm-based selections, in contrast with the strictly organised spots of a Spatial Transcriptomics slide. Critically, the technology is fully compatible with formalin-fixed paraffin-embedded samples, permitting analysis of archival disease samples and fixed biopsy material. Two independent studies utilising a NanoString immuno-oncology marker panel identified novel biomarkers that predict treatment response in melanoma [87,88].

A very recent development in the automated high-resolution large tissue cell atlasing field is the ReadCoor RC2, which utilises FISSEQ [74] combined with a stabilising matrix to map single RNA, protein, and DNA molecules *in situ*. Current panels of interest comprise ∼250 mRNAs, proteins, or DNA loci, which may be targeted combinatorially, yielding unprecedented multi-macromolecule mapping with three-dimensional single-cell resolution in a single instrument. The platform is compatible with both fresh and fixed samples up to 30 μm thick, and therefore shows promise for rich cell atlasing and comprehensive archival disease analysis alike.

One area that is expanding rapidly is technologies that allow detection and quantification of multiple molecules within a tissue, including multi-modal methods*.* For example, spatial metabolomics uses mass spectrometry to localise metabolites in tissue sections, and comparisons between this and transcriptomic or proteomic data would be valuable [89]. RNA and protein can be imaged concurrently *in situ* with some low-throughput smFISH methods like RNAscope, provided that epitopes survive the smFISH staining procedure. This is useful for determining the correlation between mRNA and protein expression, in instances where it is not possible to locate antibodies or design unambiguous probes for all of the markers needed in a multiplexed panel, or simply for better definition of specific cell types or cell boundaries by using a protein marker combined with smFISH — membrane-localised proteins make excellent cell segmentation aids. Many commercial platforms exist for assessing dozens of protein markers together in tissue sections, or RNA or protein separately, but rarely the two together. Many of the higher-throughput RNA or protein imaging approaches now being developed are also tackling the technical challenge of imaging set-up, making these techniques more accessible. Indeed many systems either incorporate an imaging system directly or are designed with software and microfluidics to automate certain microscope systems, significantly reducing hands-on time.

An ideal goal of atlasing initiatives would be not only to be able to image RNA and/or protein in thin tissue sections, but also in thicker tissue, with the goal of building 3D maps of large tissue regions or even whole organs. Significant advances have been made in tissue clearing and thick tissue imaging of proteins and mRNAs in mouse, such as CLARITY, 3DISCO, STARmap, SHANEL and others [90–93]. Methods are also developing that both clear tissue and expand it linearly to allow better visualisation of sub-cellular structure [94,95], or that can shrink large tissue volumes or even entire organisms [96]. Though low-throughput and challenging to implement, the ability to image protein and/or mRNA in thick tissue sections or even whole organs is an ideal goal of atlasing as it allows 3D reconstruction of tissues showing functional cell structures and cell–cell interactions over long distances, such as neuronal processes in the brain. Significantly, the staining of transparent human embryos and foetuses [97] and whole adult organs [91] has now been achieved.

The past decade has seen a massive technological expansion in single-cell sequencing and spatially resolved mRNA profiling. This revolution has been driven by the desire to understand the cell types that make up organisms at the molecular level with a view to better understanding basic biology and delivering translational research. Many large atlasing initiatives are in progress: what have we learnt from these early-stage ventures?

## Cell atlasing: biological insights

One of the first organisms to be ‘atlased’ was the mouse, in two main publications: the Mouse Cell Atlas [1] and Tabula Muris [2]. Between these two publications over 500 000 cells from 40 adult and foetal mouse tissues were profiled with multiple single-cell RNA sequencing (scRNAseq) technologies. The Tabula Muris revealed previously unknown roles for several genes in muscle (*Chodl*; indicating the presence of chondrocytes or cells with chondrogenic potential) and pancreas (*Neurog3* expression in somatostatin-producing delta cells; *Prss53* specifically in islet beta cells). The Mouse Cell Atlas identified the expansion of secretory alveoli in the lactating mammary gland and provided evidence of bipotent progenitors in adult murine lung. These publications also demonstrated tissue-resident mesenchymal and immune cells in several organs; observations recently extended across the murine lifespan, providing insights into ageing [98].

Other organisms being profiled with the temporal resolution are the malaria parasite *Plasmodium berghei*, the nematode worm *Caenorhabditis elegans*, and the zebrafish *Danio rerio*. Around 40% of genes in *P. berghei* currently have unknown functions, which hampers drug discovery. It is hoped that the Malaria Cell Atlas [3] will provide insight into the activity of many of these genes by comparison with genes of known function that show similar developmental expression patterns. In addition to laboratory-based parasites, the Malaria Cell Atlas also characterised ‘wild’ parasites from infected carriers at single-cell resolution, revealing their life-cycle stages. Two reports on *C. elegans* using sci-RNAseq and 10× Genomics droplet scRNAseq profiled over 130 000 cells at different stages of development [19,99]. This work identified 27 different cell types including rare neuronal lineages, and correlations with ChIP-seq data provided insights into cell type specific effects of transcription factor binding. Assessment of three different developmental time points using scRNAseq and imaging of fluorescent reporter genes demonstrated lineage convergence in several cell types and showed that many terminally differentiated cell types were generated abruptly only in the last cell division, rather than the smoother, slower differentiation paradigm previously envisaged. In zebrafish, profiling of blood cell lineage differentiation demonstrates a more gradual transition from multipotent to lineage-restricted cells [100]. This kind of temporal profiling becomes increasingly challenging as we attempt to study more complex organ systems and organisms. In mice, this can be accomplished through the use of inducible genetic reporters and techniques such as ‘Pulse-seq’ [101], but these are not feasible in humans. However, human genetic lineage tracing has now been demonstrated using mitochondrial DNA mutations, making it feasible to generate gene expression or chromatin conformation data alongside mitochondrial lineage inferences [44].

The Human Cell Atlas is one of the largest atlasing initiatives, outlined in a white paper in 2017 [102]. This brings together hundreds of scientists from around the world with the goal of identifying all cell types in the healthy human body. In addition to adult samples, the Atlas will profile developmental and paediatric samples [103] and some disease states, particularly cancer. As well as a considerable body of work being completed by individual laboratories, several co-ordinated initiatives are emerging around specific tissues. For example, LungMAP [104] and the Human Lung Cell Atlas [105] aim jointly to produce a molecular atlas of the human lung throughout foetal development, as well as in paediatric [106], and adult/ageing samples. The ‘Cell Census Network’, BRAIN initiative and others, aim to profile the brain and nervous system [107]. The Immunological Genome Project (ImmgenH) will focus on the immune system, while other groups aim to profile epithelial tissues. These atlases tend to focus heavily on high-throughput single-cell/single-nucleus RNA-sequencing techniques for initial data generation/discovery research, but are increasingly integrating proteomics and epigenetics as well as spatial sequencing/*in situ* RNA methods to understand cellular architecture at the molecular level.

**Figure 3.**
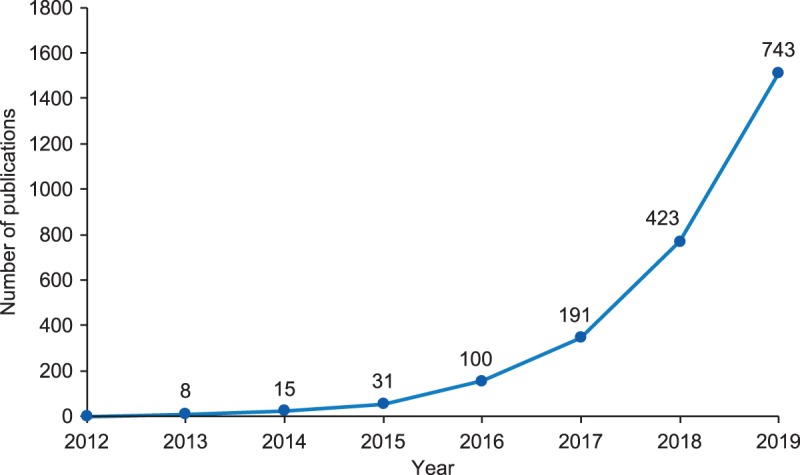
Increase in single-cell RNA sequencing publications. Total cumulative number of publications shown as a line, with total new publications per year shown above each data point. Data taken from PubMed 17/03/2020, using search term ‘single cell RNA sequencing’ in the title, abstract, or text words.

Currently, single-cell datasets have been published (or are in pre-print) regarding human lung [101,104,105,108–112], skin [22,113,114], digestive tract [115,116], female reproductive tissues including placenta [23,117–119], liver [120,121], kidney [122,123], testis [124], developing heart [125], developing brain cortex [126], developing retina [127], developing thymus [128] and cross-tissue analysis of specific cell types such as B-cells [129]. The number of human single-cell RNAseq publications is increasing rapidly (Figure 3) and already these datasets have uncovered interesting new biology.

In all tissues explored, new cell types, subtypes and cell states are being identified. Pulmonary ionocytes, which express the cystic-fibrosis gene CFTR and significantly contribute to CFTR activity, were identified in lung airways [110,112], In the decidua, new tissue-resident natural killer cell subtypes, believed to be ‘primed’ to respond to placentation in second and subsequent pregnancies, were discovered [23] and tissue-resident immune cell populations have also been identified in lung [109], digestive tract [115], and liver [120,121]. Rare progenitor cell populations are now also being uncovered, such as ‘state 0’ cells in the testis [124], progenitors in the kidney [123] and rare stem cells in the lung [108]. The single-cell analysis also reveals different cell states, such as proliferating versus quiescent cells in the lung [109]. These states can be pathogenic, as observed for mucous ciliated and epithelial cells in asthmatic lower airways [110,112], inflamed cell signatures in chronic rhinosinusitis [111], and inflammation-related signatures observed in skin epidermis, immune cell populations, endothelial cells and fibroblasts [22,113].

Cell states and transitions between differentiation stages are particularly important during development. Pseudotime analysis of single cells, rather than bulk data, lends itself to the identification of more accurate cellular trajectories. Many studies have reconstructed the developmental pathways of tissues such as kidney [123], heart [125], brain cortex [126] and retina [127]. In adult skin, temporal assessment of spatially and functionally distinct fibroblast populations demonstrates ‘priming’ of these cells that is lost with age [114]. Furthermore, comparisons between healthy and diseased tissues can infer the cell type in which the disease originates. For example, cells of the paediatric cancer Wilms tumour bear resemblance to specific foetal cells, implying aberrant early differentiation, while adult renal cell carcinoma has parallels with a subtype of proximal convoluted tubular cells [122].

On a longer timescale, comparison of cell types from different species can provide insight into evolutionary changes. Evidence for the origins of some metazoan cell types, systems and genome-regulatory mechanisms, including a potential primordial neuro-immune system in sponges [130], has been indicated by recent scRNAseq publications exploring early marine organisms [131–133]. Studies in mice indicate the suitability of mouse models of disease. For example, Travaglini et al. demonstrate 17 cell types that are either lost or gained in human compared with mouse lung tissue [109], and in heart several unique features of human versus mouse development have been identified [125]. These species differences exemplify both the utility of studying ancient organisms to better understand metazoan evolution and the value of human data for translational research.

Another valuable output of single-cell data is its ability to identify the expression of receptors and signalling molecules in individual cells, predicting cell–cell interactions and communication, as well as expression of intracellular signalling pathways. In the decidua, hormone expression is critical for cell function, for example, natural killer cells were identified with distinct chemokine profiles [23,118,119]. Cellular signalling is of course also critical during development and differentiation. Tools such as CellPhone DB, a database of receptors, ligands and their interactions, now allows searching of scRNAseq datasets to identify potential interactions [23].

Cell–cell interactions can also be inferred to an extent using imaging methods, demonstrating that cell types are physically close together. RNA and protein imaging is used extensively for validation of scRNAseq and snRNAseq data, including identification of new cell types, their locations within tissue architecture and alterations observed in disease. For example, demonstration of distinct layers of perivascular and stromal cells within the human decidua [23] and loss of excitatory *CUX2*-expressing projection neurons in upper cortical layers during multiple sclerosis by multiplexed *in situ* hybridisation [134]. Several higher-throughput imaging methods have also been used to study the mouse brain and are now being expanded into human tissue. Eng et al. [71] used SeqFISH+ to image 10 000 mRNAs in the mouse brain, detecting clear layers and cell types that correlated well with previously published scRNAseq datasets, and also analysed ligand–receptor pairs in neighbouring cells, demonstrating that gene expression is dependent on tissue context. Mouse hippocampus and human breast cancers have been profiled with *in situ* sequencing, the latter demonstrating differential gene expression within the cancerous compartment and in infiltrating lymphocytes and stroma [65,66,80]. In addition, techniques such as MERFISH allow an analysis of subcellular compartmentalisation of mRNAs [70]. Though these methods are not whole-transcriptome, they are beginning to approach a level where they can be used to spatially map tissues with little prior knowledge of cell types. Methods such as Spatial Transcriptomics, which do deliver whole-transcriptome sequencing data but not single-cell resolution, have now been used to map breast cancer, amyotrophic lateral sclerosis, pancreatic ductal carcinoma and human heart [135–138].

While spatially resolved approaches continue to develop at a rapid rate, it is still true that to date, the vast majority of atlasing publications focus on scRNAseq or snRNAseq of dissociated tissues to assess the cell types and states present. These datasets are so vast that initial published analyses can rarely explore them fully; therefore, they provide rich resources for future biological evaluation and translational research. Indeed, one of the challenges is developing the computational tools to explore these datasets (reviewed in Chen et al. [139]), particularly as single-cell multi-omics approaches advance [35,36]. Given that a cell's location within tissue is often critical for its function, spatial localisation of RNA and protein is frequently used to validate scRNAseq and snRNAseq datasets. The scale of imaging-based methods is now increasing to a point where it can be used to effectively validate massively parallel single-cell sequencing datasets. As these become commercialised and easier to run, the use of higher-throughput imaging techniques is likely to increase. Another exciting development is unbiased, spatially resolved sequencing methods with, or close to, single-cell resolution. Computational methods already exist to use scRNAseq data to deconvolute the cell types captured on spatial transcriptomics and similar platforms. It will be interesting to see if *in situ* imaging or sequencing methods reach a point where they are widely used themselves as discovery tools, mirroring the evolution of massively parallel single-cell/single-nucleus sequencing technologies.

## Cell atlasing: potential impact

Cell atlasing technologies have developed at a rapid rate over the last 10 years, with ever-widening areas of impact. They provide a comprehensive understanding of cell types, states, chromatin organisation, cell signalling networks and gene regulation mechanisms. They expand existing knowledge on how cells are organised into functional tissues. Knowledge of the molecular profile of every cell type in the human body and its location allows us to hypothesise where disease-related genetic variants may act and potential toxic side effects of new drugs. These data may enable the generation of more detailed, accurate diagnostic tests through knowledge of panels of cell type markers, their morphology and ‘normal’ spatial location. As atlasing expands to explore diseases, there is huge potential for identifying dysregulated or pathogenic cell types and states, which can be targeted with new therapeutics.

We anticipate continual drops in cost and increases in the sensitivity and breadth of applications of atlasing technologies. In particular, single-cell multi-omics technologies incorporating assessment of chromatin accessibility, epigenetics, mutational analysis and/or proteomics will expand. An area we expect to develop with particular speed is the profiling of single cells within the tissue context. As imaging technologies improve and more genes can be profiled with single-cell resolution, the scale of new data generation is likely to make another leap forwards, as tissue histology sections may contain hundreds of thousands of cells that can be profiled in parallel. Advances in tissue clearing and compatibility with single-molecule spatial methods for thick section staining will expand this into the third dimension, and the number of cells assayable into the millions, giving cell neighbourhoods and anatomical boundaries a whole new depth. Will we even start to see non- or semi-invasive highly multiplexed spatial atlasing? The ability to observe cells in their true native living context would be a phenomenal boon in understanding whole organs.

Spatial transcriptomics are likely to be coupled with methods for measuring the activation of cell signalling cascades, for example by coupling RNA data with detection of (phospho)proteins, which will then allow us to truly understand the cross-talk between neighbouring cells and interactions with surrounding structures and revolutionise our understanding of how cell communities make functional tissues. Spatially resolved technology will advance further, making it technically easier to assess the expression of thousands of RNA molecules (and other modalities) with single-cell resolution *in situ*.

**Figure 4.**
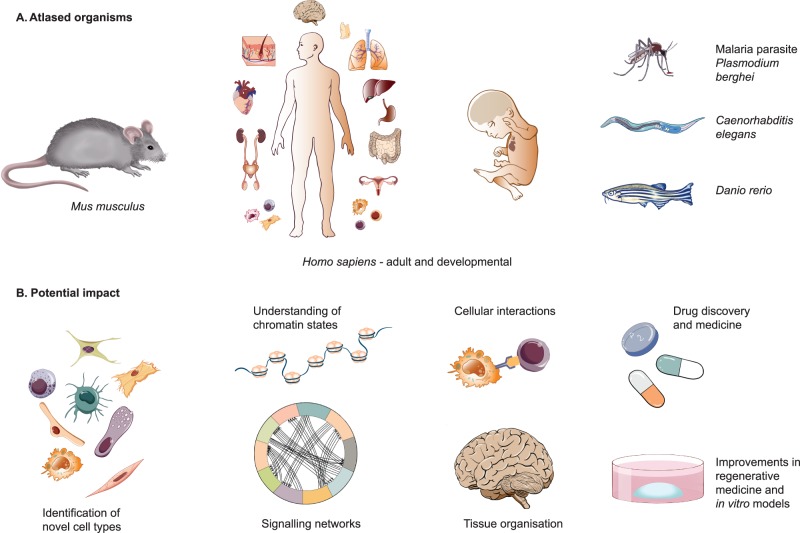
Potential impact of atlasing initiatives. Organisms for which atlasing projects are in progress. Fields that are likely to be impacted by atlasing initiatives.

Efforts in the Human Cell Atlas to collate and disseminate diverse datasets in an accessible web-browser format should make it more straightforward for scientists to interrogate these data without a need for in depth bioinformatics skills, thus expanding the utilisation of the data. Providing easy access to these vast scRNAseq and imaging datasets will hopefully produce many biologically, and potentially therapeutically, useful insights which will be functionally validated. These advances will improve our understanding of gene function, allowing assessment of the effect of knock-out versus wild type organisms with single-cell resolution and determination of the effect of inhibitors and potential drugs on whole signalling cascades in individual cells. Improvements in stem cell-derived and organoid models should feed in to this, stimulated by a better understanding of developmental processes. It will also be possible to assess the effects of drugs on organs or cell types with individual cell resolution. We will begin to understand what makes cell types that are currently hard to differentiate, different, and how that can benefit medicine. For example, understanding the full range of ion channels in different neuronal subtypes may help us to better understand pain transmission and how to treat it. We will gain insights into how our immune system functions, and how this differs throughout the body and changes with age, to better understand infection and immunity responses. A key translational area within the Human Cell Atlas is cancer, so we hope that discoveries will be made to allow us to better understand the origins of different cancers, their diversity of cell type composition and which cells are the most pathogenic. In short, atlasing initiatives are still in their infancy, and they have huge potential to impact on basic biology, technology, regenerative medicine, drug discovery and health (Figure 4), so the next decade promises many exciting advances.

## Abbreviations

ATAC: assay for transposase-accessible chromatin
FACS: fluorescence activated cell sorting
PCR: polymerase chain reaction
RCA: rolling circle amplification
scRNAseq: single-cell RNA sequencing
smFISH: single-molecule fluorescence *in situ* hybridisation
snRNAseq: single-nucleus RNA sequencing
TCR: T-cell receptor
UMI: unique molecular identifier
